# Supplementary material for: The interactome of intact mitochondria by cross-linking mass spectrometry provides evidence for coexisting respiratory supercomplexes
Source: Mol Cell Proteomics. 2017 Dec 8;17(2):216–32. doi: 10.1074/mcp.RA117.000470 (PMC5795388; doi:10.1074/mcp.RA117.000470)
Supplement: Supplemental Data [file supp_RA117.000470_133922_0_supp_23978_fzffwf.zip › spectra_annotation/mito_spectra_annotation/467-1-1-1-4-1.pdf]

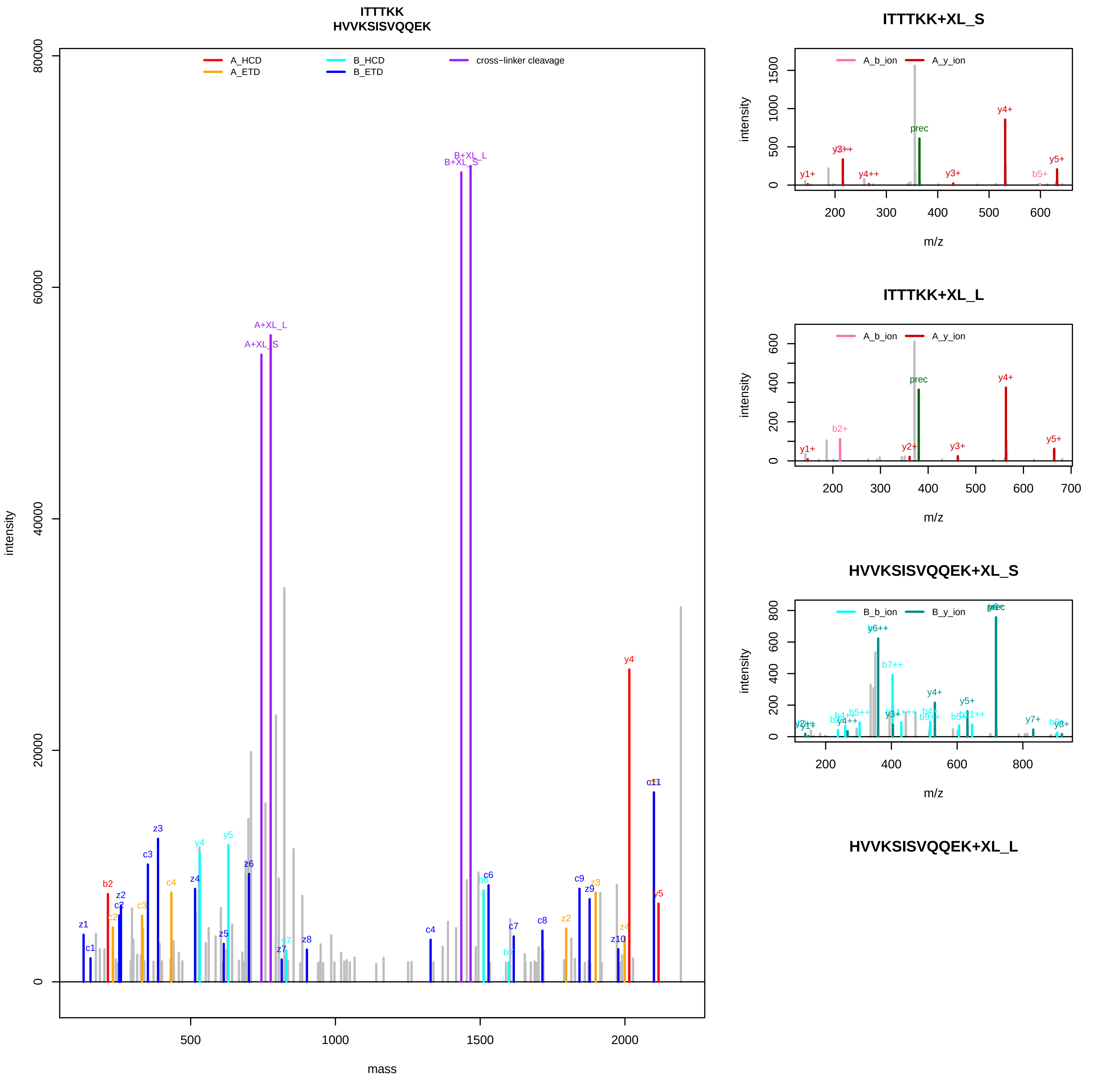
ITTTKK
HVVKSISVQQEK
A_HCD
A_ETD
B_HCD
B_ETD
cross−linker cleavage
intensity
mass
B+XL_L
B+XL_S
A+XL_L
A+XL_S
y4
y5
c11
z1
c1
b2
c2
z2
c3
z3
c4
z4
y4
z5
y5
z6
z7
y7
z8
c4
b6
c6
b7
c7
c8
z2
c9
z9
z3
z10
z4
ITTTKK+XL_S
A_b_ion
A_y_ion
y1+
y3++
y4++
prec
y3+
y4+
b5+
y5+
m/z
ITTTKK+XL_L
b2+
y2+
HVVKSISVQQEK+XL_S
B_b_ion
B_y_ion
y6++
b7++
y4+
y5+
y3+
y4++
b5++
y7+
y1+
HVVKSISVQQEK+XL_L
